# Identification of a novel nonsense mutation in the *UNC13D* gene from a patient with hemophagocytic lymphohistiocytosis: a case report

**DOI:** 10.1186/s12881-018-0600-2

**Published:** 2018-05-21

**Authors:** Xijiang Hu, Dongling Liu, Xiwen Jiang, Bo Gao, Changying Chen

**Affiliations:** 1 Wuhan Children’s Hospital (Wuhan Maternal and Child Healthcare Hospital), Tongji Medical College, Huazhong University of Science and Technology, Wuhan, 430016 Hubei China; 2 School of Nursing, Zhengzhou University, Zhengzhou, 450052 Henan China; 3 DaAn Gene Co., Ltd. Of Sun Yat-sen University, The Medicine and Biological Engineering Technology Research Center of the Ministry of Health, Guangzhou, Guangdong China; 4 Department of Laboratory Medicine, Taihe Hospital, Hubei University of Medicine, Shiyan, Hubei China

**Keywords:** *UNC13D*, Nonsense mutation, HLH, Amplicon sequencing, Molecular diagnosis

## Abstract

**Background:**

Hemophagocytic lymphohistiocytosis (HLH) is a heterogeneous and potentially fatal disease that presents symptoms of persistent fever, splenomegaly and cytopenia. Primary HLH is identified as an autosomal recessive disorder with causative genes including *HPLH1*, *PRF1*, *UNC13D*, *STX11* and *STXBP2*.

**Case presentation:**

Here, we reported an 8-month-old female patient with compound heterozygosity in the *UNC13D* gene. The patient, who presented typical symptoms, was diagnosed with HLH based on HLH-2004 guidelines. High-throughput amplicon sequencing for the full-length exon, including a 5 bp padding region and 6 HLH-related genes, was performed to identify the pathogenic mutations in this patient. In all, 9 heterozygous variations were detected, namely, 7 nonpathogenic SNPs, one nonsense mutation (NM_199242.2:c.2206C > T, p.Gln736X), and one splicing mutation (NM_199242.2:c.2709 + 1G > A). These two mutations were considered pathogenic according to previous studies and functional prediction. A two-generation pedigree analysis based on Sanger sequencing was performed to confirm the result.

**Conclusion:**

Compound heterozygosity in the *UNC13D* gene was identified in trans and considered a causative mutation in a female patient with HLH. The nonsense mutation (NM_199242.2:c.2206C > T, p.Gln736X) was novel in cases of HLH. Our data expand the spectrum of HLH-related mutations in China and demonstrate the potential of high-throughput amplicon sequencing in the diagnosis of HLH.

## Background

Hemophagocytic lymphohistiocytosis (HLH) is a heterogeneous and potentially fatal disease that presents with symptoms of persistent fever, splenomegaly and cytopenia. The first case was reported in 1952 by Farquhar et al. [1]; uncontrolled proliferation of activated macrophages and T lymphocytes are hallmarks of this syndrome [2]. Two types of HLH were described: a primary and a secondary form [3]. Primary HLH, also termed familial hemophagocytic lymphohistiocytosis (FHL), is an autosomal recessive genetic disorder with five major causative genes, namely, *HPLH1*, *PRF1*, *UNC13D*, *STX11* and *STXBP2* (Table 1) [4]. The incidence of FHL was estimated to approximately 1:100000 and 1:50000, respectively, according to two independent studies [5, 6]; however, worldwide, the incidence of FHL is still unclear. The secondary HLH, or acquired HLH, is mostly caused by the strong immunological activation of the immune system (e.g., severe infection of virus) rather than genetic mutations, but it may also develop during malignancies. It is difficult to distinguish these two types of HLH from each other, which necessitates an approach to differential diagnoses.

**Table 1:** Subtypes of familial hemophagocytic lymphohistiocytosis and X-linked lymphoproliferative syndrome

Subtype	OMIM ID	Causative gene	HGNC ID	Cytogenetic location	Inheritance
FHL1	267700	*HPLH1* ^a^	HGNC:23824	9q21.3 - q22	AR
FHL2	603553	*PRF1*	HGNC:9360	10q22.1	AR
FHL3	608898	*UNC13D*	HGNC:23147	17q25.1	AR ^b^
FHL4	603552	*STX11*	HGNC:11429	6q24.2	AR
FHL5	613101	*STXBP2*	HGNC:11445	19p13.2	AR ^b^
XLP1	308240	*SH2D1A*	HGNC:10820	Xq25	XLR
XLP2	300635	*XIAP*	HGNC:592	Xq25	XLR

*AR* autosomal recessive, *XLR* X-linked recessive

^a^ The HGNC locus types of *HPLH1* is phenotype only, meaning mapped phenotypes where the causative gene has not been identified (SO:0001500)

^b^ Both FHL3 and FHL5 could be caused by homozygous or compound heterozygous mutations in causative gene

Here, we performed high-throughput amplicon sequencing to detect mutations in whole exons of 6 HLH-related genes for an 8-month-old female patient, who was diagnosed with HLH based on HLH-2004 guidelines [7]. As a result, 9 heterozygous variations, namely, 7 SNPs, one nonsense mutation (c.2206C > T) and one splicing mutation (c.2709 + 1G > A), in the *UNC13D* gene were detected. The nonsense mutation was reported in HLH for the first time. The compound heterozygous mutations in the *UNC13D* gene were considered causative mutations for the patient based on previous studies and functional predictions. A two-generation pedigree analysis showed that they were inherited from the parents. The result suggests great potential of high-throughput amplicon sequencing in the diagnosis of HLH.

## Case presentation

This study was approved by the ethics committee on the use of human subjects at Wuhan Children’s Hospital. Informed consent from the patient’s parents was obtained before collecting blood samples. The patient and her parents are all Han Chinese from Hubei province of China.

**Fig. 1:**
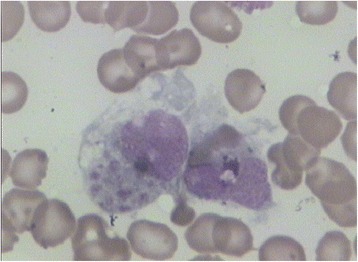
The bone marrow examination. Phagocytosis was clearly observed without any evidence of malignancy

This 8-month-old female patient was admitted to Wuhan Children’s Hospital after 2 days of fever, with a temperature measured at 39.0 °C on regular examination. Blood analysis showed low platelets (PLT, 45 × 10^9^/L) and low hemoglobin (Hb, 7.7 g/100 mL). Low NK cell activity (4.45%) and plasma albumin (18.2 g/L) were also observed. Moderate splenomegaly was revealed by ultrasound examination. The bone marrow examination suggested hemophagocytosis (Fig. 1), and no features of malignancy were observed. Since six of the eight criteria of HLH-2004 were fulfilled [7], this patient was diagnosed with HLH. To confirm the subtype of HLH and to investigate causative mutations in this patient, high-throughput amplicon sequencing and two-generation pedigree analysis were performed.

Genomic DNA was purified from peripheral blood mononuclear cells (PBMCs) with a commercial kit purchased from TIANGEN Biotech (Beijing, China). Multiple PCR primers were designed for 6 genes using Ion AmpliSeq™ Designer (Table 1); the *SH2D1A* and *XIAP* genes were included for distinguishing X-linked lymphoproliferative syndrome (XLP), which has a strong resemblance to HLH [8]. All exons of these genes were covered by 198 amplicons, including 5 bp of padding region. High-throughput amplicon sequencing was performed using an ION S5XL genetic analyzer (Thermo Fisher Scientific, MA, USA). VariantCaller V1.0 was used to detect mutations from sequencing data.

As a result, 9 heterozygous variations were detected, namely, 5 synonymous mutations, one missense mutation, one noncoding region mutation, one nonsense mutation and one splicing mutation (Table 2). All mutations were considered to be benign according to the dbSNP database except for the nonsense mutation NM_199242.2:c.2206C > T and the splicing mutation NM_199242.2:c.2709 + 1G > A. The splicing mutation c.2709 + 1G > A has been reported in patients with HLH with compound heterozygous mutations in the *UNC13D* gene [9, 10], it was predicted to be broken wild-type donor site and most probably affecting splicing, according to the human splicing finder (HSF, version 3.1). However, the nonsense mutation c.2206C > T, which resulted in the introduction of a premature stop codon and produced truncated mRNA (p.Gln736X), was reported in HLH for the first time. The compound heterozygosity was considered to be causative in this case, based on functional alteration analysis and previous reports.

**Table 2:** Detected variations in the patient with HLH

Mutation	Position in reference genome GRCh37	Amino acid change	Type	Frequency (%)	Zygosity	ID in dbSNP	Allele Frequency in ExAC
*PRF1*:c.900C > T	NC_000010.10:g.72358577	p.His300His	Synonymous	49	Heterozygous	rs885822	0.6357
*UNC13D*:c.3198A > G	NC_000017.10:g.73824121	p.Glu1066Glu	Synonymous	53	Heterozygous	rs7210574	0.4844
*UNC13D*:c.2599A > G	NC_000017.10:73827205	p.Lys867Glu	Missense	50	Heterozygous	rs1135688	0.3666
*UNC13D*:c.1977C > T	NC_000017.10:73831016	p.Thr659Thr	Synonymous	43	Heterozygous	rs2290770	0.0318
*UNC13D*:c.888G > C	NC_000017.10:73836162	p.Pro296Pro	Synonymous	49	Heterozygous	rs3744026	0.1525
*UNC13D*:c.279C > T	NC_000017.10:73839137	p.Pro93Pro	Synonymous	53	Heterozygous	rs3744007	0.0518
*XIAP*:c.*12A > G	NC_000023.10:g.123041043	–	Non-coding region	51	Heterozygous	rs28382740	0.2695
*UNC13D*:c.2709 + 1G > A	NC_000017.10:73826658	–	Splicing	49	Heterozygous	not applicable	not applicable
*UNC13D*:c.2206C > T	NC_000017.10:73830498	p.Gln736X	Nonsense	48	Heterozygous	not applicable	not applicable

**Fig. 2:**
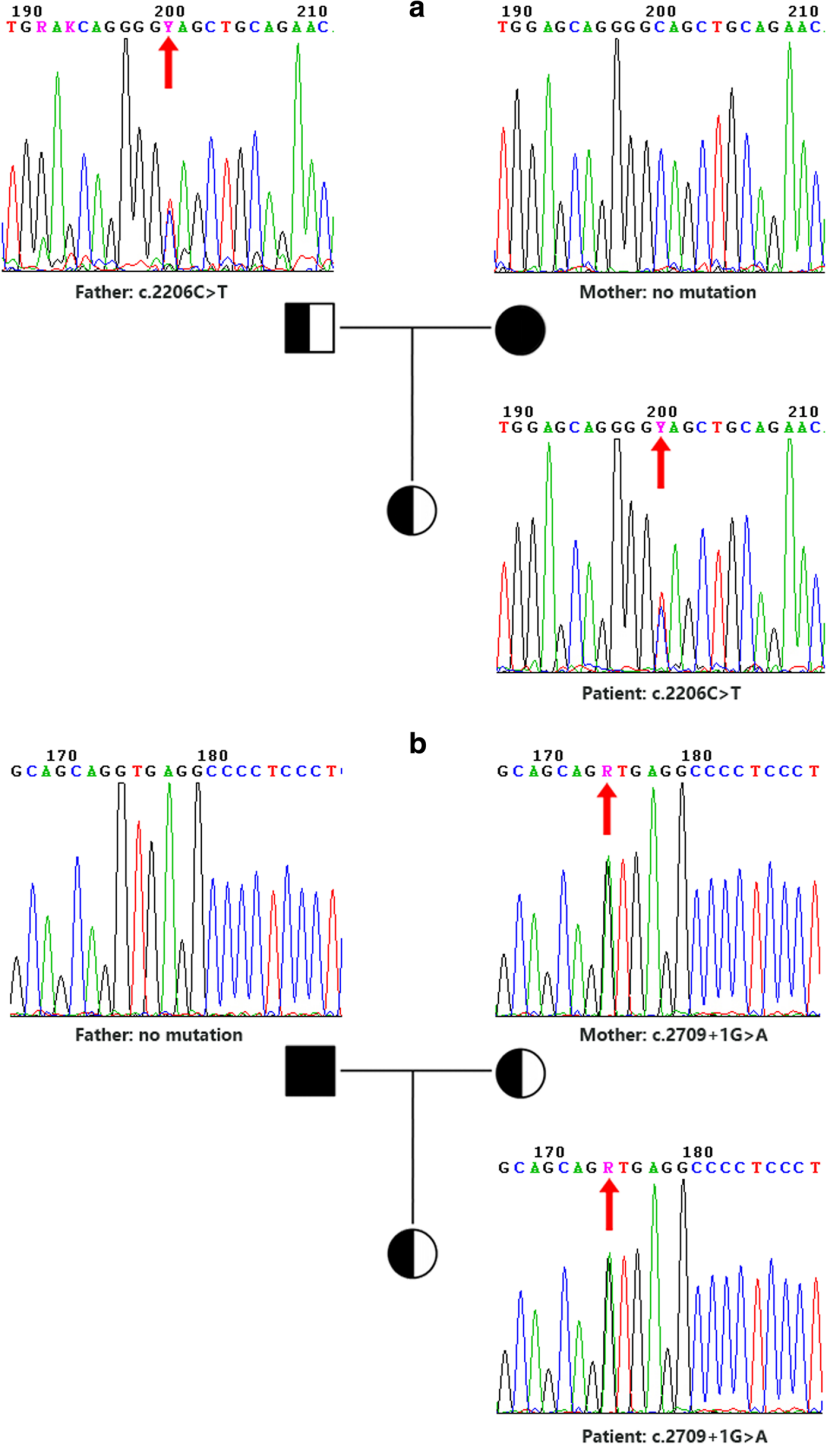
Two-generation pedigree analysis for two heterozygous mutations in the *UNC13D* gene. **a** Novel nonsense mutation (c.2206C > T) in *UNC13D*-exon24; **b** Reported splicing mutation (c.2709 + 1G > A) in *UNC13D*-exon28. The position of the mutation is marked with a red arrow. The results showed that the nonsense mutation was inherited from father and the splicing mutation was inherited from mother. Zygosity was indicated by letters of degenerate bases (Y: C/T, R: A/G)

Both mutations were confirmed by Sanger sequencing, and a two-generation pedigree analysis was performed with the parents’ samples. The results showed these two mutations were inherited from the father and mother (Fig. 2), presenting a typical autosomal recessive mode of inheritance.

## Discussion and conclusions

In this case, an 8-month-old female patient was admitted to Wuhan Children’s Hospital and diagnosed with HLH based on clinical features, including persistent fever, moderate splenomegaly, low PLT and Hb, decreased NK-cell activity, and hemophagocytosis with no evidence of malignancy. Compound heterozygous mutations consisting of a nonsense mutation (c.2206C > T) and a splicing mutation (c.2709 + 1G > A) in the *UNC13D* gene were identified in this patient using high-throughput amplicon sequencing. A two-generation pedigree analysis based on Sanger sequencing confirmed the results and revealed a typical autosomal recessive inheritance mode in this family.

HLH is a heterogeneous disease with two major conditions and five subtypes; additionally, many other conditions can lead to the clinical picture of HLH, such as malignancies, rheumatoid disorders, and XLP [7]. Molecular diagnosis based on genetic tests provide a powerful tool for differential diagnoses of HLH, which is important for appropriate treatment. Bianca et al. reported an approach for the genetic screening of patients with HLH using targeted high-throughput sequencing of 12 related genes, which resulted in a diagnosis in 22 of 58 patients in the prospective cohort [11]. Further in-depth investigations of the phenotype-genotype relationship of HLH are required to develop better sequencing panels for diagnosis.

The *UNC13D* gene, which encodes a protein involved in the cytotoxic activity of T lymphocytes, was confirmed as a causative gene of familial HLH type 3. Pathogenic mutations in the *UNC13D* gene are very common in patients with HLH, as 17–19% of FHL patients from Turkey and Germany, 89% from Korea, and 30% from Japan were identified to have pathogenic mutations in the *UNC13D* gene [12–14]*.* Compound heterozygosity in the *UNC13D* gene was also reported, especially in the splicing site [9, 10]. However, there has been no genetic investigation on a substantial number of HLH patients in China. In this case, we identified the causative mutations of this patient in 24 h, using our customized panel which was designed automatically with online tool and purchased from Thermo Fisher Scientific (MA, USA). Average base coverage depth of the 55 kb target region was 1070-fold, 96.44% of total base was sequenced more than 100-fold. The concordance rate of amplicon sequencing and sanger sequencing is 100% in this case. The cost of this approach is about 1/5 to 1/10 of whole exome sequencing. The application of high-throughput amplicon sequencing would be helpful to diagnose genetic disease with an efficient and cost-saving way.

In conclusion, compound heterozygosity consisting of a nonsense mutation (c.2206C > T) and a splicing mutation (c.2709 + 1G > A) in the *UNC13D* gene was identified in a patient with HLH using high-throughput amplicon sequencing. Both mutations were confirmed by Sanger sequencing and were inherited from the parents. Our results expand the spectrum of *UNC13D* mutations in Chinese patients with HLH and demonstrate the potential of high-throughput amplicon sequencing in the diagnosis of heterogeneous genetic disorders.

## Abbreviations

FHL: Familial hemophagocytic lymphohistiocytosis
Hb: Hemoglobin
HLH: Hemophagocytic lymphohistiocytosis
PBMC: Peripheral blood mononuclear cells
PLT: Platelets
XLP: X-linked lymphoproliferative syndrome
